# A cut above

**DOI:** 10.7554/eLife.25000

**Published:** 2017-02-15

**Authors:** Chongsheng He, Roberto Bonasio

**Affiliations:** Epigenetics Institute and the Department of Cell and Developmental Biology, Perelman School of Medicine, University of Pennsylvania, Philadelphia, United States; Epigenetics Institute and the Department of Cell and Developmental Biology, Perelman School of Medicine, University of Pennsylvania, Philadelphia, United Statesrbon@mail.med.upenn.edu

**Keywords:** transcription factors, chromatin, in situ profiling, DNA sequencing, chromatin mapping, *S. cerevisiae*, Human

## Abstract

A new technique called CUT&RUN can map the distribution of proteins on the genome with higher resolution and accuracy than existing approaches.

**Related research article** Skene P, Henikoff S. 2017. An efficient targeted nuclease strategy for high-resolution mapping of DNA binding sites. *eLife* **6**:e21856. doi: 10.7554/eLife.21856

The genome is regulated by thousands of proteins and mapping the interactions between these proteins and the genome is crucial in many areas of biology. However, the mapping process is complicated by the fact that eukaryotic genomes are normally packaged inside a macromolecular complex called chromatin, which is found in the cell nucleus. The basic building block of chromatin is a structure called a nucleosome, which consists of about 150 base pairs of DNA wrapped around proteins called histones (Campos and Reinberg, 2009). The most popular technique for mapping protein–DNA interactions is called ChIP, which is short for chromatin immunoprecipitation.

Although a number of variations on ChIP have been developed over the years (Zentner and Henikoff, 2014), the original method – called crosslinked ChIP or X-ChIP – is still the most widely used. In X-ChIP the protein-DNA interactions are frozen in place (by using formaldehyde to form crosslinks between them; Figure 1A) and the chromatin is fragmented by sonication and made soluble. Antibodies are then used to physically separate the chromatin fragments that contain the protein of interest from those that do not, so that the DNA in these fragments can be identified. In native ChIP, the freezing-in-place (crosslinking) step is skipped and the chromatin is fragmented by an enzyme called micrococcal nuclease (MNase; Figure 1B). The DNA obtained by X-ChIP or native ChIP can be identified using microarrays (ChIP-chip) or deep sequencing techniques (ChIP-seq; Johnson et al., 2007). The final results are maps spanning the entire genome and studded with peaks and valleys that represent the presence and absence of a given protein at each genomic locus.

**Figure 1.**
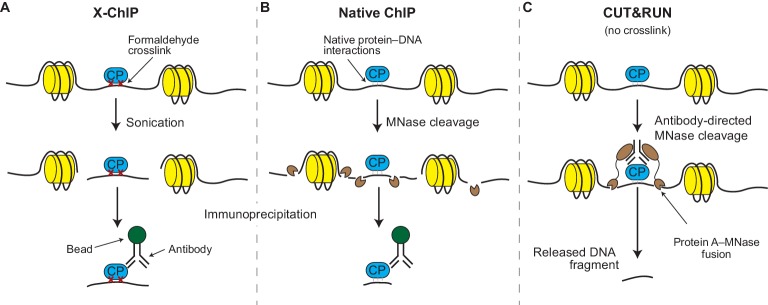
X-ChIP, native ChIP, and CUT&RUN. (**A**) In X-ChIP, cells are first crosslinked (red crosses) with formaldehyde to freeze the interactions between the DNA (black line) and a chromatin-binding protein of interest (CP; blue). Sonication fragments the chromatin and makes it soluble. Antibodies are used to recognize the protein–DNA fragments, which are then ‘pulled’ out of the solution using antibody-binding beads, in a process called immunoprecipitation. The histones are shown in yellow. (**B**) In native ChIP, chromatin is fragmented and solubilized by treating cells with an enzyme called micrococcal nuclease (MNase; small brown shapes). The natural affinity of the protein for its DNA target keep them together during the immunoprecipitation process. (**C**) In CUT&RUN, antibodies direct the activity of the MNase enzyme to ensure that chromatin cleavage happens close to the protein of interest. A protein called protein A (brown ovals) binds the MNase enzyme to the antibody. The resulting small DNA fragments can be isolated as they diffuse out of the nuclei.

Crosslinking-based ChIP techniques, especially ChIP-seq, are now widely used to study gene regulation and they have been employed in a number of large-scale studies, such as the ENCODE project (ENCODE Consortium, 2012) and the NIH Roadmap Epigenomics project (Roadmap Epigenomics Consortium, 2015). Thousands of ChIP-seq datasets are available in public databases and there are very few papers in the fields of transcription, gene regulation, and epigenetics that do not contain at least one ChIP-seq experiment.

However, ChIP-seq suffers from a number of limitations, including poor resolution, suboptimal signal-to-noise ratio, and a tendency for false positives. These problems are caused by the chemicals used to crosslink the chromatin, the strong forces used to fragment it during sonication, and the detergents used to make the fragments soluble (Jain et al., 2015; Teytelman et al., 2013). Now, in eLife, Peter Skene and Steven Henikoff of the Fred Hutchinson Cancer Research Center report that they have developed a new technique called ‘cleavage under targets and release using nuclease’ (CUT&RUN) that has the potential to overcome the limitations of X-ChIP (Skene and Henikoff, 2017).

CUT&RUN is similar to native ChIP in that it uses the same enzyme, MNase, to fragment the DNA (Figure 1C). However, unlike native ChIP, the antibodies are not used to physically separate wanted from unwanted chromatin fragments (which is one of the sources of noise in ChIP); instead, CUT&RUN uses the antibodies to guide the cutting activity of the MNase enzyme to the protein of interest while the latter is still bound to intact chromatin. This means that only nearby DNA is cut into small fragments; because of their size, these fragments of DNA float out of the nuclei (leaving the rest of the uncut genome behind) and can be identified by deep sequencing. Therefore, a significant advantage of CUT&RUN is that it does not require the use of strong chemicals or sonication and that the nuclease only cuts the DNA in the region of interest, thus minimizing the noise from unwanted chromatin regions. Skene and Henikoff borrowed this idea from an older technique called chromatin immunocleavage (ChIC; Schmid et al., 2004) but adapted it to native nuclei and modernized it for the sequencing age.

To test the performance of CUT&RUN, Skene and Henikoff created maps of well-known proteins that bind the genome in yeast and human cells and compared these to maps produced by ChIP-seq. The maps obtained through CUT&RUN had a higher dynamic range and recovered a larger fraction of ‘true positives’ (sites containing short DNA sequences recognized by each protein). Skene and Henikoff were also able to modify CUT&RUN to study poorly soluble complexes by extracting all the DNA from the nuclei and only sequencing fragments below a certain size limit, which were specifically generated by the antibody-guided MNase enzyme. Due to lower sample and sequencing requirements, CUT&RUN might be applicable to a number of real-world situations, where cost and sample availability are often an issue.

As a new technique, CUT&RUN will require further evaluation to determine whether it could replace (or at least complement) ChIP-seq and its variants at the same scale. For example, it is not clear if CUT&RUN can be applied to samples in which it is not possible to isolate the nuclei of the cells; this could include some fresh tissue samples and many samples that have been fixed and then frozen. Moreover, like X-ChIP, CUT&RUN has the disadvantage that in addition to cleaving DNA in the target region, the nuclease also cleaves DNA that is far away from the target region in sequence space, but close in real space because of the three-dimensional structure of the DNA. To distinguish between these direct and indirect targets, Skene and Henikoff compared CUT&RUN maps with those obtained with their native ChIP protocol (Kasinathan et al., 2014), which only detects direct targets. However, this two-pronged approach can be labor intensive and CUT&RUN would be even more useful if it could be modified to only detect DNA directly bound to the protein of interest.

Nevertheless, CUT&RUN is a welcome addition to the arsenal of techniques in the fast-evolving field of functional genomics and we look forward to the new insights that it might facilitate in the future.
